# An annotated near-complete sequence assembly of the *Magnaporthe oryzae* 70-15 reference genome

**DOI:** 10.1038/s41597-025-05116-3

**Published:** 2025-05-07

**Authors:** Hang-yuan Cheng, Li-ping Jiang, Yue Fei, Fei Lu, Shengwei Ma

**Affiliations:** 1 Institute of Genetics and Developmental Biology, Chinese Academy of Sciences, Beijing, 100101 P. R. China; 2 University of Chinese Academy of Sciences, Beijing, 100049 P. R. China; 3 Yazhouwan National Laboratory, Sanya, Hainan 572024 P. R. China

**Keywords:** Agricultural genetics, Fungal genomics

## Abstract

*Magnaporthe oryzae* is a devastating fungal pathogen that causes substantial yield losses in rice and other cereal crops worldwide. A high-quality genome assembly is critical for addressing challenges posed by this pathogen. However, the current widely used MG8 assembly of the *M. oryzae* strain 70-15 reference genome contains numerous gaps and unresolved repetitive regions. Here, we report a complete 44.82 Mb high-quality nuclear genome and a 35.95 kb circular mitochondrial genome for strain 70-15, generated using deep-coverage PacBio high-fidelity sequencing (HiFi) and high-resolution chromatin conformation capture (Hi-C) data. Notably, we successfully resolved one or both telomere sequences for all seven chromosomes and achieved telomere-to-telomere (T2T) assemblies for chromosomes 2, 3, 4, 6, and 7. Based on this T2T assembly, we predicted 12,100 protein-coding genes and 493 effectors. This high-quality T2T assembly represents a significant advancement in *M. oryzae* genomics and provides an enhanced reference for studies in genome biology, comparative genomics, and population genetics of this economically important plant pathogen.

## Background & Summary

Filamentous plant pathogens, including fungi and oomycetes, pose widespread and severe threats to global crop production and food security. These devastating pathogens are estimated to account for approximately 10–23% of agricultural production losses annually^1–3^. Rice blast, caused by the fungal pathogen *Magnaporthe oryzae*, represents the most destructive disease of rice (*Oryza sativa*) worldwide, resulting in annual yield losses of 10–30%, an amount sufficient to feed 60 million people^4,5^.

Given its significant threat to the economy and food security, *M. oryzae* became a milestone in fungal genomics as the first fungus to have its genome sequenced (MG8 version from *M. oryzae* strain 70-15) in 2005^6^. Since then, the advent of advanced sequencing technologies has accelerated fungal genomics research, with the *M. oryzae* strain 70-15 MG8 genome version serving as a primary reference for comparative genomic studies. As of 2024, more than 350 genome assemblies of different *M. oryzae* strains have been generated and are now available in The National Center for Biotechnology Information (NCBI) databases. Some strains have even achieved T2T level assembly quality^7^. However, the widely-used reference genome of *M. oryzae* strain 70-15 remains to be updated since its initial release. This MG8 version, generated through Sanger sequencing, contains substantial gaps and missing repeat regions due to the technical limitations of the technology. To overcome these limitations and enhance our understanding of *M. oryzae* biology, we employed an integrated approach combining deep-coverage HiFi sequencing and high-resolution Hi-C technologies to generate a comprehensive genome assembly of strain 70-15. Our assembly yielded a complete 44.82-Mb nuclear genome and a 35.95-kb circular mitochondrial genome.

**Fig. 1:**
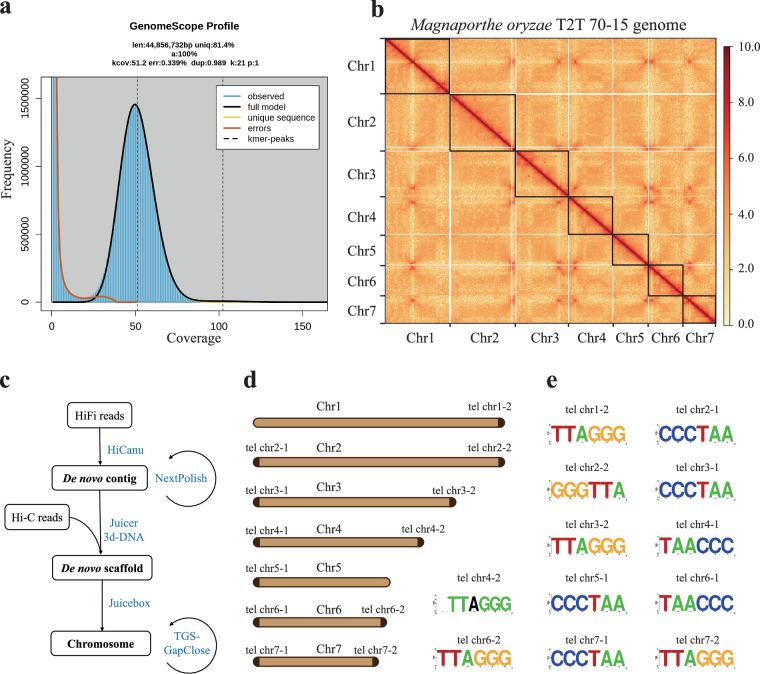
*M. oryzae* T2T 70-15 genome survey, assembly and evaluation. (**a**) Genome survey analysis of *Magnaporthe oryzae* 70-15. The genome size of *M. oryzae* 70–15 was estimated to be ~ 44.85 Mb using the 21-mer depth distribution of the 30X Illumina sequence. (**b**) Chromosome interaction heatmaps (100-kb resolution) are based on Hi-C signals. The black boxes represent chromosomal regions. (**c**) Pipeline for assembly of *Magnaporthe oryzae* 70-15 T2T genome. (**d**) and (**e**) showed the chromosome characteristics with telomere structure and the repetitive pattern of corresponding telomeres, respectively. Dark brown blocks indicated that telomere repeats were detected at the ends of chromosomes.

**Fig. 2:**
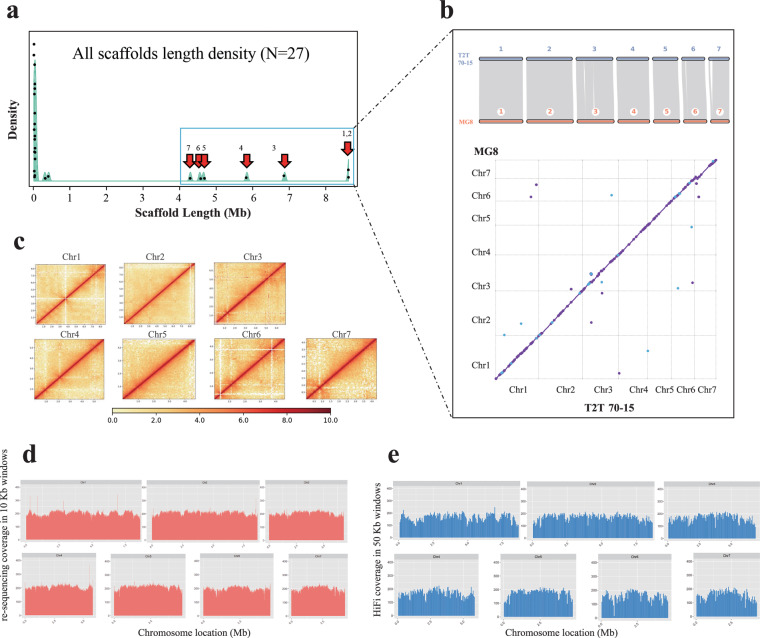
Collinear analysis and phasing assessment for T2T genome assemblies. (**a**) Out of a total of 27 assembled scaffolds, the longest 7 scaffolds were selected as pseudochromosomes. The black dot represents a scaffold with a length corresponding to the X-axis. The green histogram represents the distribution density of scaffolds with a specific length range. (**b**) These seven pseudochromosomes showed a good collinearity with the seven MG8 chromosomes at the nucleic acid (right bottom) and protein levels (right top), respectively. (**c**) The seven-chromosome hicmaps (50-kb resolution) supported the chromosome assembly with good internal continuity. The mapping depth distribution of HiFi and NGS reads were further used for phasing assessment of genome assemblies. (**d**) NGS reads mapping depth distribution based on 10 kb sliding windows. (**e**) HiFi reads mapping depth distribution based on 50 kb sliding windows.

**Fig. 3:**
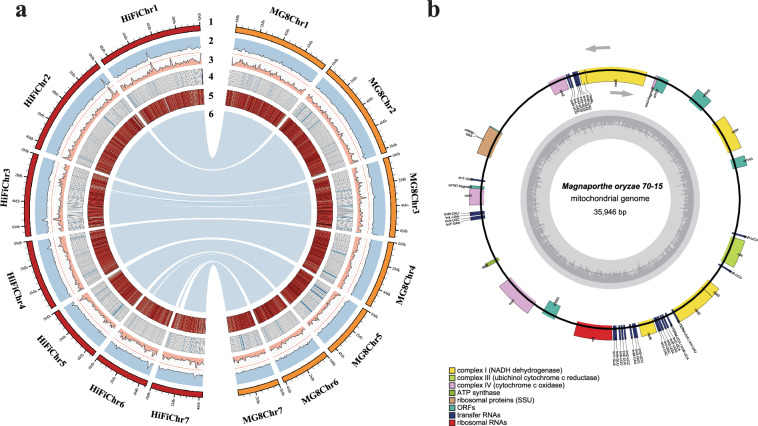
Genome features of *M. oryzae* 70-15 nuclear genome and mitochondrial genome. (**a**) A representative genome circos comparing T2T 70-15 and MG8 genome. For the circle map, track 1 on the periphery illustrates assembled T2T 70-15 (left, dark brown) and published MG8 genome (right, light brown). Track 2-6 represent the GC ratio (outside-in orientation), the percentage of repeats (in-outside orientation), the non-coding loci density, the protein-coding genes density, and collinearity regions between T2T 70-15 and MG8 chromosomes, respectively. A sliding window of 10Kb was used to count the number of genes. (**b**) Genome features of *M. oryzae* 70-15 mitochondrial genome. Outer track depicts a circular illustration of the mitochondrial genome carrying genes.

The genome size of *M. oryzae* 70-15 was estimated to be approximately 44.8 Mb using k-mer frequency analysis based on about 220 × coverage (9.92 Gb) Illumina paired-end short clean reads (Fig. 1a). This estimated size is 9% larger than the MG8 version, implying the presence of unresolved genomic regions in 70-15. A total of 173× coverage (7.79 Gb) of PacBio HiFi reads and 186× coverage (7.36 Gb) of Hi-C sequencing reads were generated to assemble a high-quality *M. oryzae* 70-15 genome. Using an assembly workflow described in method, the final assembly comprised 27 scaffolds with a genome size of 44.82 Mb and the N50 value of 6.85 Mb (Table 1). The seven longest and gap-free scaffolds were assigned into seven pseudochromosomes (Figs. 1b, 2a), with a combined size of 43.46 Mb, representing 97% of estimated genome size. High collinearity was observed between these seven pseudochromosomes and their counterparts in the MG8 genome version (Figs. 2b, 3a). Moreover, the 35.95-kb mitochondrial genome was identified within the remaining scaffolds by BLASTN using the mitochondrial genome from *M. oryzae* strain P131 as a query^7^.

**Table 1:** Summary statistics of the *M. oryzae* T2T 70-15 genome assembly and comparison to the MG8 assembly.

Scaffold features	T2T 70-15	MG8
Total assembly size (Mb)	44.82	41.03
Anchored size (Mb)	43.46	40.49
GC content (%)	51.07	51.61
Repeat content (%)	17.97	11.78
**Contiguity**		
N50 length (Mb)	6.85	6.61
N90 length (Mb)	4.29	4.13
L50 number	3	3
L90 number	7	6
Gaps in chromosomes	0	157
**Completeness**		
BUSCO (%)	97.6	97.5
LAI	32.79	15.73
**Correctness**		
Read mapping (%)	99.87	90.79
Error rate	2.0 × 10^−6^	1.4 × 10^−5^

Additionally, we manually searched for the previously reported 150-bp repeat sequence (CCCTAA/TTAGGG)n, a known signature of *M. oryzae* chromosome telomeres^8^. Double-ended telomere repeat unit was detected on gapless chromosomes 2, 3, 4, 6, and 7, meaning that these five chromosomes were thus resolved in a T2T manner. The remaining two chromosomes were also detected to have a single-ended telomere repeat unit (Fig. 1d,e). Finally, we achieved the T2T golden reference genome for *M. oryzae* strain 70-15, which was named as T2T 70-15.

Based on this high-quality T2T assembly, we updated the nuclear genome repeat elements annotation. The percentage of total repeat elements annotated in the T2T 70-15 genome reached 16.93%, significantly higher than the MG8 version. The T2T 70-15 genome assembly was superior in both the number and length of various repeat elements (Table 2), indicating that the T2T assembly had a more complete repeat resolution. This improvement in the annotation of repeat elements, especially retroelements and transposons, will facilitate the exploration of the mechanisms underlying the genetic diversification and epigenetic control of effectors^9^.

**Table 2:** Characteristics of repeat elements in the *M. oryzae* T2T 70-15 and MG8 assemblies.

Elements	T2T 70-15			MG8		
	Number	Length (bp)	Percentage of genome	Number	Length (bp)	Percentage of genome
Total	19,793	7,358,534	16.93%	17,671	4,770,601	11.78%
Retroelements	2,114	5,226,394	12.02%	1,337	2,953,443	7.29%
LINEs	244	732,291	1.68%	223	655,623	1.62%
LTR elements	1,720	4,428,191	10.19%	1,114	2,297,820	5.67%
*Ty1/Copia*	484	1,463,764	3.37%	205	497,563	1.23%
*Gypsy*/DIRS1	1,228	2,963,879	6.82%	909	1,800,257	4.45%
DNA transposons	879	962,508	2.21%	653	870,093	2.15%
Small RNA	105	32,990	0.08%	81	8,404	0.02%
Simple repeats	12,881	478,739	1.11%	12,204	447,226	1.11%
Low complexity	1,746	77,857	0.18%	1,640	72,577	0.18%
Unclassified	2,061	577,664	1.33%	1,756	418,858	1.03%

We then focused on the protein-coding genes and carried out combined annotation methods to annotate the T2T 70-15 genome. A total of 5.07 Gb of RNA sequencing was generated from strain 70-15 grown in oat medium. Combined with 12 published *M. oryzae* transcriptome under a range of growth conditions^10,11^, we annotated a total of 12,100 protein-coding genes in the T2T 70-15 nuclear genome (Table 3). Simultaneously, the mitochondrial genome was annotated by MFannot^12^, including genes encoding 14 standard fungal core, ribosomal subunits and 27 tRNA genes (Fig. 3b). Additionally, as filamentous plant pathogens have a large repertoire of effector proteins that facilitate their infection of the host, we thus annotated the effectors encoded by the *M. oryzae* T2T 70-15 based on both domain prediction^3^ and machine learning predictions^13^. We eventually obtained 493 high-confidence effectors^14^.

**Table 3:** Updated annotation of the T2T *M. oryzae* strain 70-15 genome.

Chromosome features		
Size (Mb)	43.46	
GC content (%)	51.07	
Complete BUSCOs (%)	97.6	
Repeat content (%)	16.9	
**Protein-coding gene**		
Gene number	12,100	
Mean gene length (bp)	1,624	
Mean exon length (bp)	574	
Mean exon number per gene	2.50	
**Non-coding loci**	**Number**	**Total length (bp)**
rRNAs	67	53,588
tRNAs	285	23,340
ncRNAs	29	6,210

## Methods

### Strain material, nucleic acid extraction and sequencing

*M. oryzae* strain 70–15 was first incubated at 28 °C in the dark for 3 days after monospore isolation before being incubated at 28 °C in the light for about 10 days. The hyphae and spores were collected and grown in liquid LB medium at 28 °C in the dark with shaking at 220 rpm. After 5 days, the hyphae were collected by filtration for the construction of PacBio and Illumina sequencing libraries.

The genomic DNA of harvested strain 70-15 was prepared by the CTAB method and followed by purification with QIAGEN® Genomic kit (Cat#13343, QIAGEN). The DNA degradation and contamination of the extracted DNA was monitored on 1% agarose gels. DNA purity was then detected using NanoDrop™ One UV-Vis spectrophotometer (Thermo Fisher Scientific, USA). DNA concentration was further measured by Qubit® 4.0 Fluorometer (Invitrogen, USA). SMRTbell target size libraries were constructed for sequencing according to PacBio’s standard protocol (Pacific Biosciences, CA, USA) using 15 kb preparation solutions. Briefly, a total amount of 15 µg DNA per sample was used for the DNA library preparations. The genomic DNA sample was sheared by g-TUBEs (Covaris, USA) according to the expected size of the fragments for the library. Single-strand overhangs were then removed, and DNA fragments were damage repaired, end repaired and A-tailing. Then the fragments ligated with the hairpin adaptor for PacBio sequencing. And the library was treated by nuclease with SMRTbell Enzyme Cleanup Kit and purified by AMPure PB Beads. Target fragments were screened by the BluePippin (Sage Science, USA). The SMRTbell library was then purified by AMPure PB beads, and Agilent 2100 Bioanalyzer (Agilent technologies, USA) was used to detect the size of library fragments. Sequencing was performed on a PacBio Sequel II instrument in CCS mode with Sequencing Primer V2 and Sequel II Binding Kit 2.0 in Grandomics.

To construct the Hi-C library and obtain sequencing data, the harvested strain 70-15 was cut into pieces and vacuum infiltrated in nuclei isolation buffer supplemented with 2% formaldehyde. Crosslinking was stopped by adding glycine and additional vacuum infiltration. Fixed tissue was then grounded to powder before re-suspending in nuclei isolation buffer to obtain a suspension of nuclei. The purified nuclei were digested with 100 units of DpnII and marked by incubating with biotin-14-dATP. Biotin-14-dATP from non-ligated DNA ends was removed owing to the exonuclease activity of T4 DNA polymerase. The ligated DNA was sheared into 300–600 bp fragments, and then was blunt-end repaired and A-tailed, followed by purification through biotin-streptavidin-mediated pull down. Finally, the Hi-C libraries were quantified and sequenced using the MGI-2000 platform.

Libraries for Illumina paired-end genome sequencing were constructed using Truseq Nano DNA HT Sample preparation Kit (Illumina, USA) following the standard manufacturers protocol. Approximately 1.5 µg genomic DNA per sample was fragmented by sonication to an average size of 350 bp, DNA fragments were then blunted with an A-base overhang and ligated to sequencing adapters for Illumina sequencing with further PCR amplification. At last, PCR products were purified by AMPure XP beads (Beckman Coulter) and libraries were analyzed for size distribution by Agilent2100 Bioanalyzer and quantified using real-time PCR. After that, the library was sequenced on the Illumina NovaSeq. 6000 platform with a paired-end sequencing strategy.

Total RNA was extracted by grinding tissue in TRIzol reagent TIANGEN/CTAB-LiCl method on dry ice and processed following the protocol provided by the manufacturer. Sequencing libraries were generated using TruSeq RNA Library Preparation Kit (Illumina, USA) following standard protocol. Briefly, about 1 µg RNA per sample was used and enriched from total RNA using oligo(dT)-attached magnetic beads. The firs strand cDNA was synthesized with random primer and M-MLV Reverse Transcriptase, and then second strand cDNA synthesis was followed by using DNA Polymerase I and RNase H. The synthesized cDNA was end-repaired, A-tailing added and ligated to the sequencing adapters. The cDNA fragments were selected by AMPure XP beads (Beckman Coulter) to an average size of 150–200 bp and amplified by PCR with Phusion High-Fidelity DNA polymerase, Universal PCR primers and Index Primer. At last, PCR products were purified with AMPure XP Beads (Beckman Coulter, USA) and library quality was assessed on the Agilent Bioanalyzer 2100 system. After that, the library was sequenced on the Illumina NovaSeq. 6000 platform.

### *De novo* assembly of the T2T *M. oryzae* 70-15 genome

The primary T2T genome assembly of strain 70-15 was generated from two assemblers (Hicanu v.2.2 and Hifiasm v.0.16)^15,16^, using ‘-l0’ and ‘genomeSize = 40 m useGrid = false -pacbio-hifi’ parameters, respectively. The contigs assembled by Hicanu were selected as final contigs based on a comprehensive evaluation of contiguity, completeness, and correctness (Table S1). Potential misassemblies were corrected using NextPolish v.1.4.1^17^ with two rounds of polishing with HiFi long reads and four rounds of polishing with paired-end short reads, with the setting ‘task = best rerun = 3 max_depth 100’ in the parameter config file. The pseudo-chromosomes of *M. oryzae* 70-15 were then assembled with Hi-C reads, using Juicer v.1.6^18^ and 3D-DNA v.180922^19^, sequentially. Possible assembly errors were manually corrected using JuicerBox v.1.9.8^20^. The final genome assembly was optimized and supplemented in TGS-GapCloser v.1.0.3^21^ using HiFi reads, with the setting command ‘-minmap_arg ‘-x asm20’–tgstype pb’. The last 3 gaps were closed by manual extension using the HiFi reads. Purge_dups was used to automatically identify and remove haplotigs and heterozygous duplications (parameters: −2 -f 1 -T cutoffs)^22^. The intrachromosomal Hi-C contact matrix was generated with HiC-Pro v.3.1.0 and visualized with HiCPlotter^23^. The complete pipeline for the assembly of the *M. oryzae* T2T 70-15 genome is summarized in Fig. 1c.

### Genome assessment and visualization

Basic genome assembly statistics were obtained with QUAST v5.2.0^24^ and assembly-stats v.1.0.1. The assembly completeness of genic regions was evaluated using the sordariomycetes_odb10 dataset (https://busco-data.ezlab.org/v5/data/lineages/sordariomycetes_odb10.2020-08-05.tar.gz) of BUSCO v.5.4.2^25^, with default parameters. To assess the correctness of the new genome assembly, Illumina paired-end short sequencing reads generated by this study were mapped to the assembly with BWA-mem v.0.7.17 and SAMtools v.1.15^26^. Merqury v1.3^27^ was used to compute consensus quality (QV) and k-mer completeness. Mummer v.3.23^28^ and MCscanX jcvi v.1.3.3^29^ were then applied to analyze and visualize genome collinearity with default parameters. Circos v.0.69.8^30^ was used to visualize the T2T 70-15 genome assembly as a circular plot and to compare it to the MG8 genome assembly^6^.

### Genome annotation

The total number of repeat regions in the whole genome was identified using RepeatModeler v.2.0.3 and RepeatMasker v.4.1.5^31^. The LTR retrotransposons were annotated and the LAI was estimated using LTR_Finder v.1.07^32^ and LTR_retriever v.2.9.4^33^, respectively (parameters: -D 20000 -d 1000 -L 700 -l 100 -p 20 -C -M 0.9). Prediction of non-protein coding RNA genes like tRNA, rRNA, and ncRNA was performed based on INFERNAL (cmscan) v.1.1.4 and Rfam 14.9^34^.

The annotation of protein-coding genes was based on *ab initio* gene predictions, transcriptome-based annotation, and homologous protein predictions. For *ab initio* gene predictions, AUGUSTUS v.3.4.0^35^ was deployed using trained species sets, with the ‘–species = magnaporthe_grisea’ parameter. GeneMark-ES^36^ was also used for *ab initio* gene prediction using default settings and the fungi mode. For homologous proteins-based predictions, protein sequences were collected from published chromosome-level genome assemblies of the *M. oryzae* taxon. After eliminating redundant sequences using CD-HIT v4.8.1^37^, genes encoding non-redundant proteins were annotated on the assembly via miniport V.0.13-r248^38^. For transcript-based predictions, 12 published RNA-seq datasets were downloaded via the NCBI and ENA browsers (NCBI BioProject accession PRJNA52817; ENA browser Project: PRJEB45007). These RNA-seq data represented almost all the physiological states of *M. oryzae*, constituting a reliable and comprehensive complement of transcripts, namely heat (42 °C), cold (4 °C), light, darkness, high salinity (500 mM NaCl), and at 8 h, 16 h, 24 h, 48 h, 72 h, 96 h, and 144 h post-infection of a host plant (Table S2). HISAT2 v.2.2.1^39^ was used to perform splice site–aware alignment of paired-end RNA-seq reads to the assembled genomes, with the ‘–dta’ parameter. The transcripts were then assembled using StringTie v.2.2.1^40^. TransDecoder v.5.5.0^41^ was applied to predict coding regions according to the above assembled transcripts. All annotation results predicted above were integrated in the EVidencemodeler v.2.1.0 pipeline^42^.

### Variation calling

The whole-genome sequencing reads generated by this study were mapped to the *M. oryzae* 70-15 MG8 and the T2T genome assemblies using BWA-mem v.0.7.17 with default parameters. Alignments were sorted with SAMtools v.1.10 and duplicates were removed with Picard (http://broadinstitute.github.io/picard/). Variants were identified using GATK v.4.1.8.1^43^. The following thresholds were applied: QD < 20.0; MQ < 40.0; FS > 3. In order to avoid the errors by the misalignment, we used the PopDepth pipeline to remove outliers with ultrahigh or low depth^44^. Only biallelic SNPs were retained as high-confidence variants. The comparative structural variant analysis was carried out using SyRi v.1.7.0^45^.

### Prediction of effectors

Integrated prediction for effectors was applied by the following three rules: (1) the presence of a signal peptide predicted by SignalP v.6.0^46^; (2) the absence of a transmembrane domain beyond the first 60 amino acids predicted by TMHMM v.2.0^47^; and (3) positive identification as a secreted protein candidate, predicted by effectorP v.3.0^13^. The proteins at the intersection of the three above criteria were considered as high-confidence candidate effectors^14^.

## Data Recodes

All raw data and assembly results have been submitted to the NCBI database under BioProject PRJNA1210831. The raw genomic sequencing data are available at NCBI Sequence Read Archive database under accession number SRR32814542, SRR32815023, SRR32814693, and SRR32814013^48^. The assembled genome was deposited at NCBI under accession number JBMMUB000000000^49^. The gene, repeat and ncRNA annotation are available at Figshare^14^. The raw sequencing data and genome assembly can also be retrieved at The National Genomics Data Center under BioProject accession PRJCA034974.

## Technical Validation

### Quality assessments of the assembly completeness

We assessed the completeness of our 70-15 genome assembly by calculating the benchmarking universal single-copy orthologs (BUSCO) score, using a sordariomycetes gene base. We obtained a BUSCO score of 97.6% for complete single-copy genes. Additional busco genes were included in the HiFi genome, bringing a slight increase in conserved gene pool integrity. Notably, compared to the MG8, our 70-15 genome assembly showed a higher long terminal repeat (LTR) assembly index (LAI). The LAI of our 70-15 genome assembly was 32.8, thus well above the value of 20 used to classify a genome as a golden reference^50^, indicating that the new assembly should exhibit high integrity for LTR sequences (Table 1). Furthermore, we utilized merqury to evaluate the genome assembly using both short-read and long-read sequencing data. The computed QV scores were 47.93 (short-read) and 50.41 (long-read), while the k-mer completeness values reached 99.36% and 99.60%, respectively. These metrics consistently demonstrate the high completeness of the assembled genome.

### Quality assessments of the assembly correctness

To validate the correctness of the new genome assembly, we mapped Next-Generation Sequencing (NGS) short reads to the assembled chromosomes and achieved a mapping rate of 99.87% (Table 1) with a uniform mapping depth distribution (Fig. 2d). The coverage of NGS and HiFi reads in chromosomal regions was over 99.86% (Fig. 2d,e; Fig. S1). We also called SNPs and INDELs on the T2T 70-15 genome to check the correctness through mapping the NGS sequencing data to itself. The possible assembly error rate represented by the variation rate was around 2 × 10^−6^, which is about seven times lower than that obtained for the MG8 version (Fig. S2). Moreover, we performed structural variation (SV) analysis and identified 22 translocations and inversions between the two genomes using SyRI (Table S3). Subsequently a detailed examination of these 22 translocations and inversions was carried out through visualization of HiFi reads mapping. The results revealed that 20 SV loci (±10 kb) in the newly assembled genome exhibited excellent HiFi read coverage, with multiple HiFi reads supporting the accuracy of the corresponding regions in the new assembly (Figs. S3, S4). The remaining two inversions were located around the original genomic gaps, which may introduce scaffolding errors in T2T assembly. By contrast, 13/22 SV regions were found to contain gaps within a 10 kb proximity in the MG8 reference genome, implying a higher frequency of error introduction. T2T assembly demonstrates substantial improvement by rectifying potential mis-joins within the previous reference genome.

### Quality assessments of the assembly contiguity

The assembled T2T 70-15 genome exhibits no gaps in chromosomal regions, demonstrating excellent genome contiguity. To further assess the contiguity and accuracy of our 70-15 genome assembly, we collected public long-read sequencing *M. oryzae* species genomes that assembled into the chromosome level (Table S4): B71, Br48, T3, and ZM1-2 isolated from bread wheat (*Triticum aestivum*)^51,52^; EA18, P131, and TRG2 isolated from rice^7,53^; LpKY97 isolated from the perennial ryegrass (*Lolium perenne*)^54^; MZ5-1-6 isolated from finger millet (*Eleusine coracana*)^55^; and TF05-1MC7 isolated from tall fescue (*Lolium arundinaceum*). A genome-wide collinearity analysis revealed that the genomes of closely related species within the *M. oryzae* species complex exhibit good collinearity. Previously reported large chromosomal translocation^55^ at the boundary between chromosome 1 and chromosome 6 can also be captured (Fig. S5).

## Supplementary information

Supplementary Table S1,Supplementary Table S2, Supplementary Table S3, Supplementary Table S4

Supplementary Figure

## Data Availability

The published software used in this work is listed in the Methods section. If no detailed parameters were mentioned for the software, default parameters were used.
