# Supplementary material for: An annotated near-complete sequence assembly of the Magnaporthe oryzae 70-15 reference genome
Source: Sci Data. 2025 May 7;12:758. doi: 10.1038/s41597-025-05116-3 (PMC12059122; doi:10.1038/s41597-025-05116-3)
Supplement: Supplementary file 2 — Supplementary Figure [file 41597_2025_5116_MOESM2_ESM.pdf]

Figure S1

a

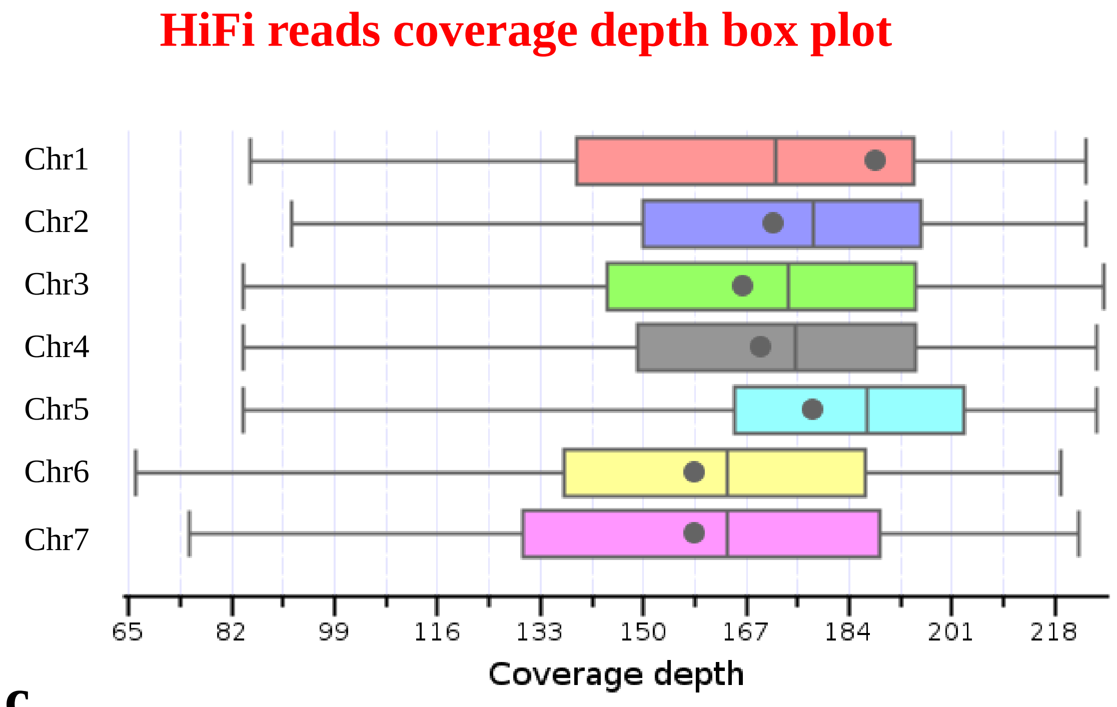

b

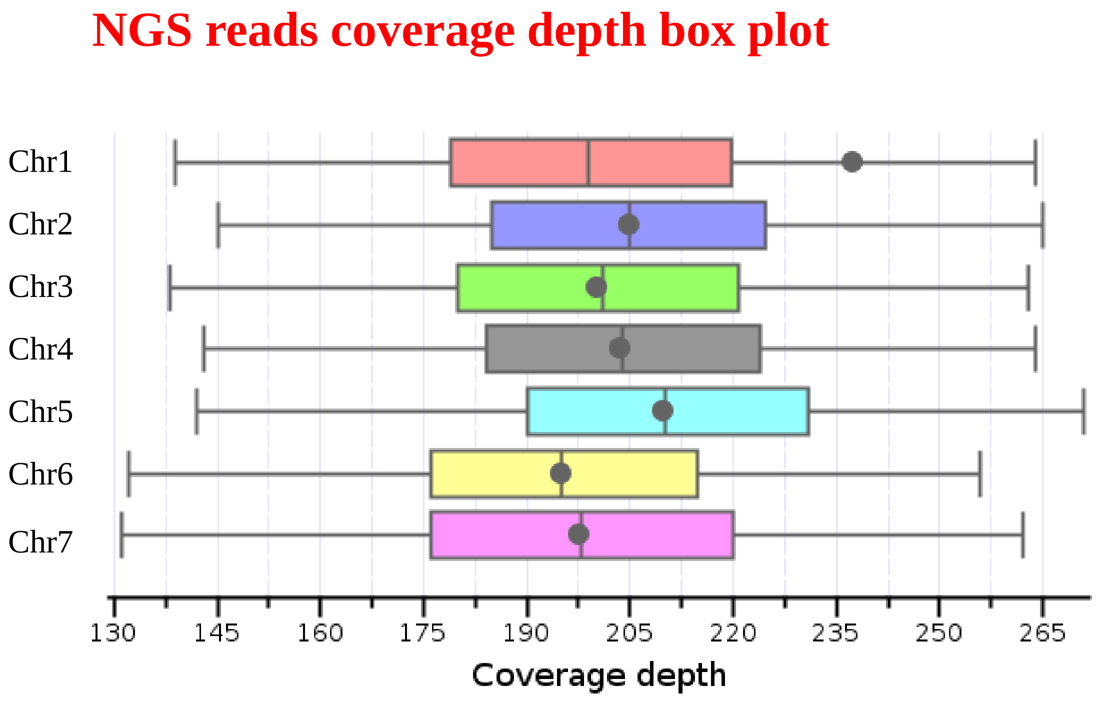

c

Chromosome	NGS reads mapping		HiFi reads mapping	
	Coverage (%)	Avg depth	Coverage (%)	Avg depth
Chr1	100.00	238.72	100.00	188.04
Chr2	100.00	205.63	100.00	171.30
Chr3	99.98	202.19	100.00	167.78
Chr4	100.00	204.83	100.00	169.98
Chr5	100.00	210.90	100.00	178.13
Chr6	99.98	195.75	99.86	158.12
Chr7	99.98	198.45	99.97	158.15

Fig. S1 The mapping coverage and average depth of assembled 7 *M. oryzae* HiFi 70-15 chromosomes

The mapping coverage and average depth of every chromosome in *M. oryzae* T2T 70-15 genome were examined by HiFi and NGS reads. (**a**) Box plot showed the range of HiFi reads coverage depth in each chromosome. (**b**) Box plot showed the range of NGS reads coverage depth in each chromosome. (**c**) The corresponding mapping coverage percentage (%) and average depth statistics of NGS and HiFi reads on the T2T 70-15 genome. The average depth here was calculated by single nucleotide base.

Figure S2

a

Compare the chromosome size and correctness		
Parameters	HiFi 70-15	MG8
Chromosome total size (Mb)	43.46	40.49
Chromosome 1	8.62	7.979
Chromosome 2	8.62	8.320
Chromosome 3	6.85	6.607
Chromosome 4	5.82	5.547
Chromosome 5	4.68	4.490
Chromosome 6	4.58	4.134
Chromosome 7	4.29	3.416
SNPs	28	320
Indels	62	278
Error rate	$2.0*10^{-6}$	$1.4*10^{-5}$

b

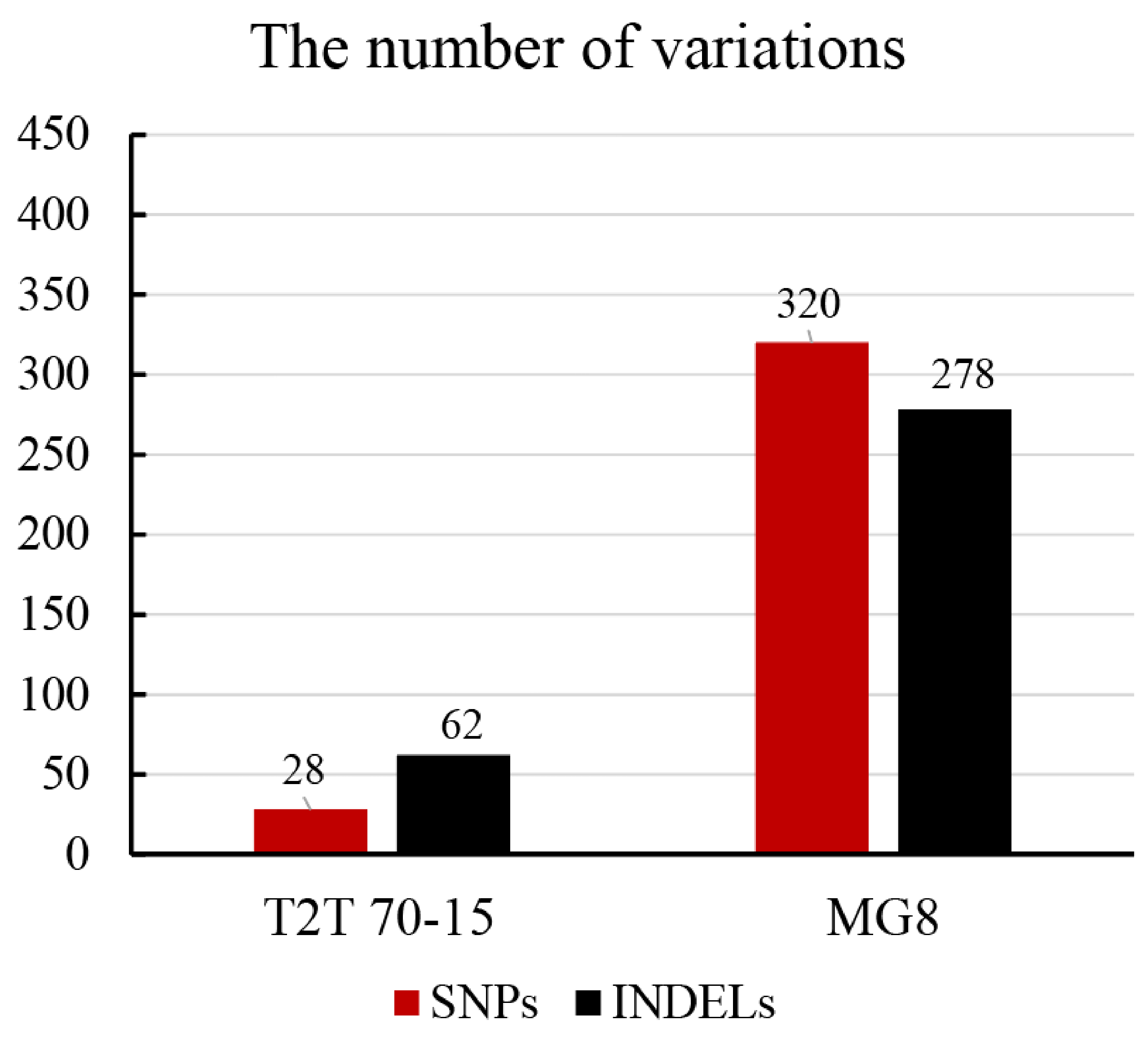

Fig. S2 Comparison of the chromosome correctness between T2T 70-15 and MG8.

The SNPs and INDELs were called and filtered (For SNPs: DP<5.0, MQ<20.0, FS>80.0, QD<2.0, GT=0/1; For INDELs: QD<2.0, FS>200.0, GT=0/1) to check the correctness of the assembled *M. oryzae* genome. (**a**) The table compared the chromosome size and correctness between T2T 70-15 and MG8. (**b**) The bar graph showed the number of variations in T2T 70-15 and MG8 genome. We obtained 97 SNPs and 35 INDELs on the T2T 70-15 genome. The number of variations on the T2T 70-15 genome was reduced by ~ 7 times compared to the MG8 version. The variation rate was calculated by dividing the sum of SNPs and INDELs by the total chromosome size (bp).

Figure S3

TRANS 1: HiFi70-15_Chr7:720,738-744,314

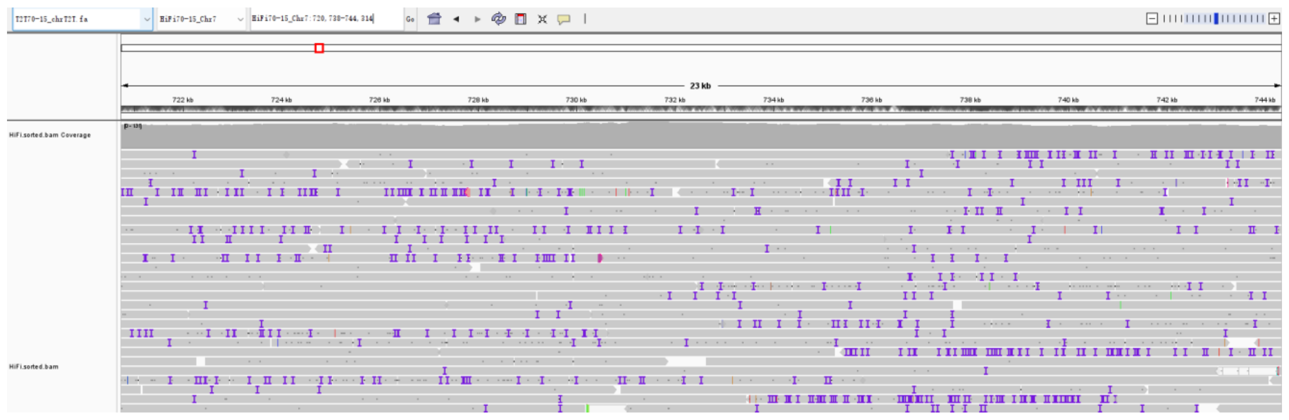

TRANS 2: HiFi70-15_Chr7:711,584-737,212

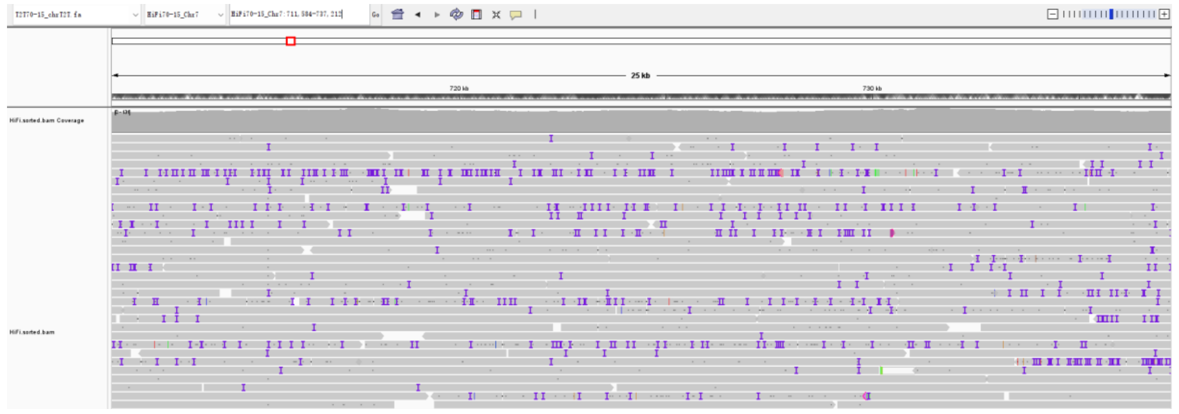

TRANS 3: HiFi70-15_Chr7:835,150-860,555

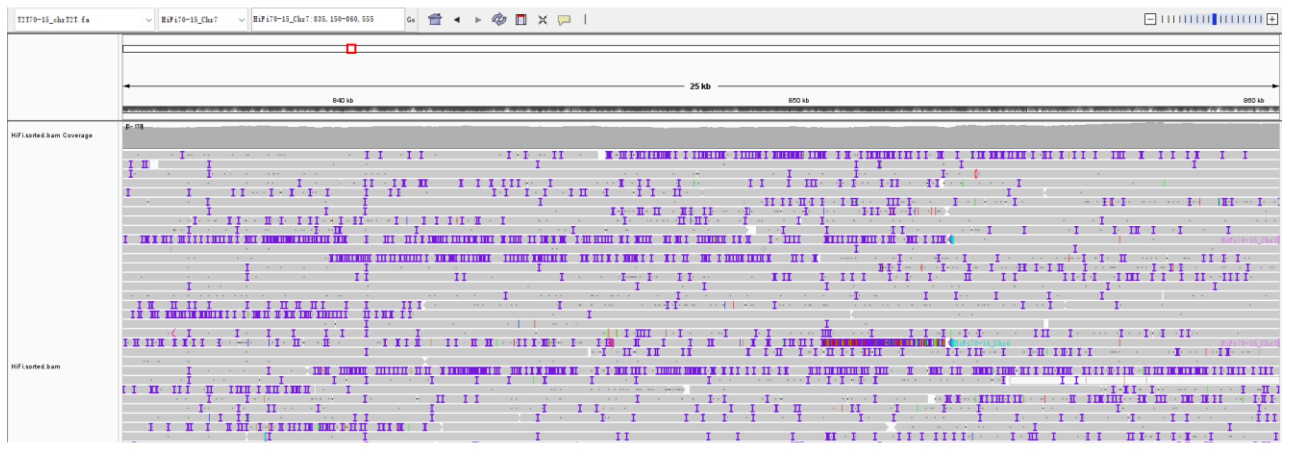

TRANS 4: HiFi70-15_Chr7:724,900-746,473

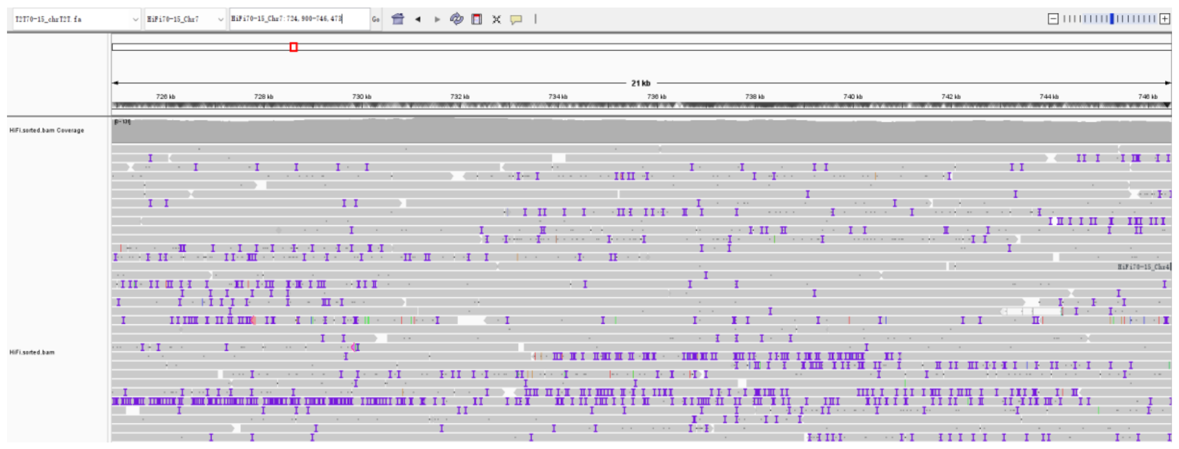

TRANS 5: HiFi70-15_Chr7:509,428-533,543

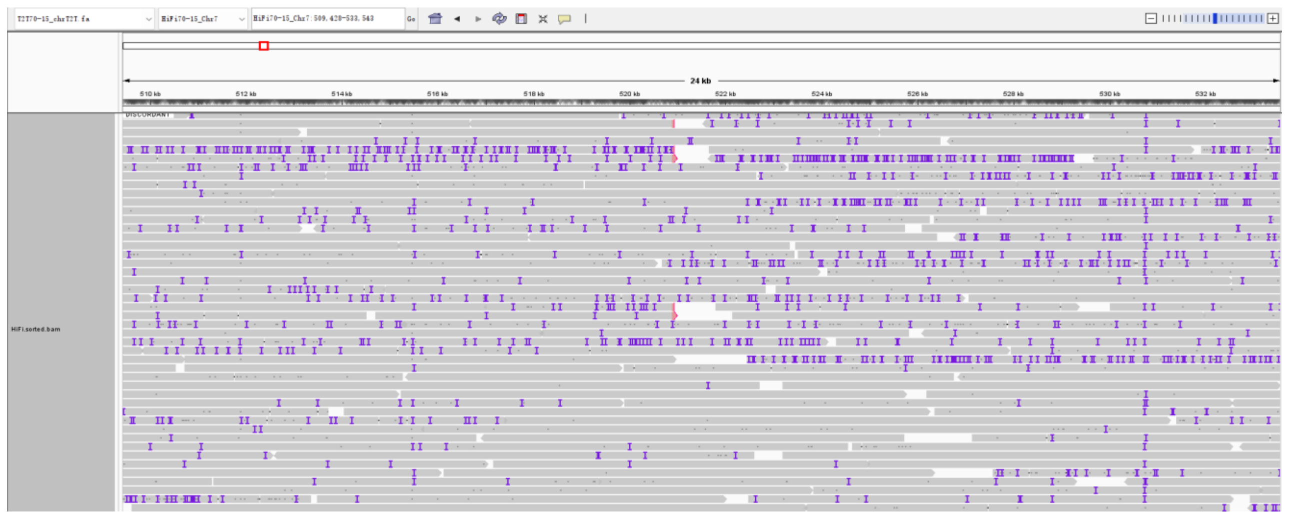

TRANS 6: HiFi70-15_Chr7:563,150-583,668

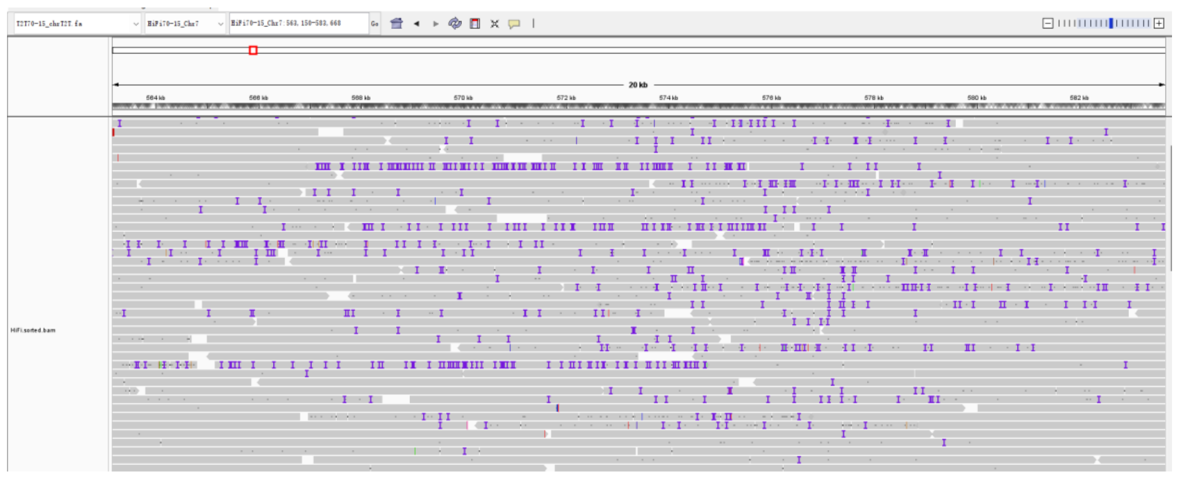

TRANS 7: HiFi70-15_Chr7:569,674-593,906

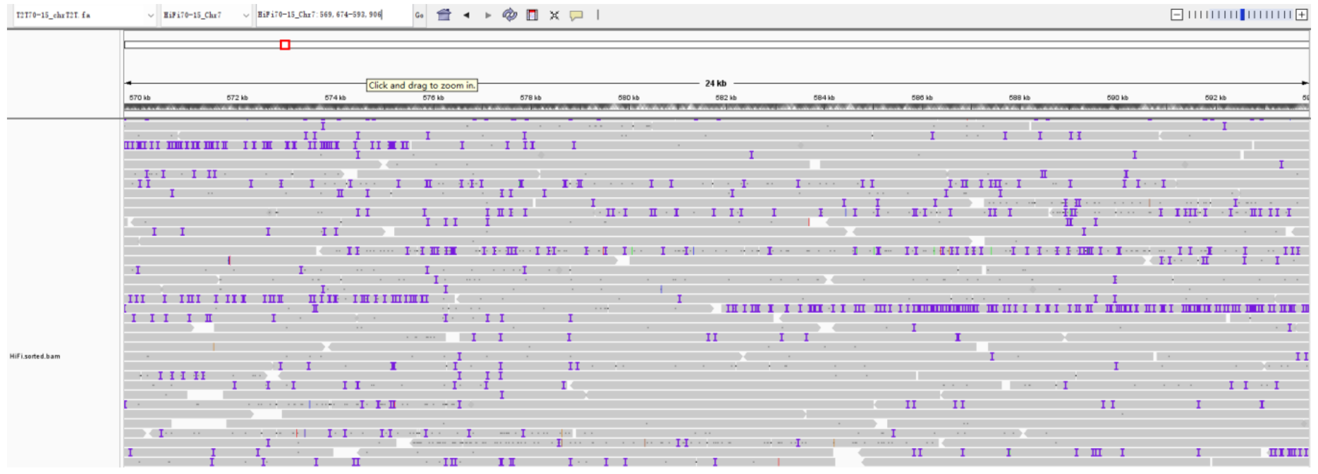

TRANS 8: HiFi70-15_Chr7:711,557-734,589

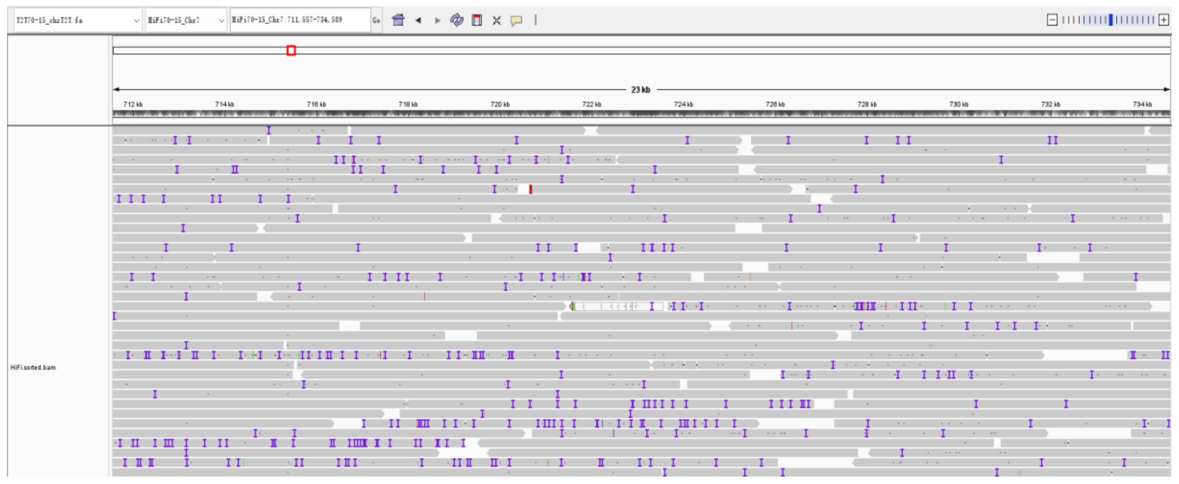

TRANS 9: HiFi70-15_Chr7:777,371-799,223

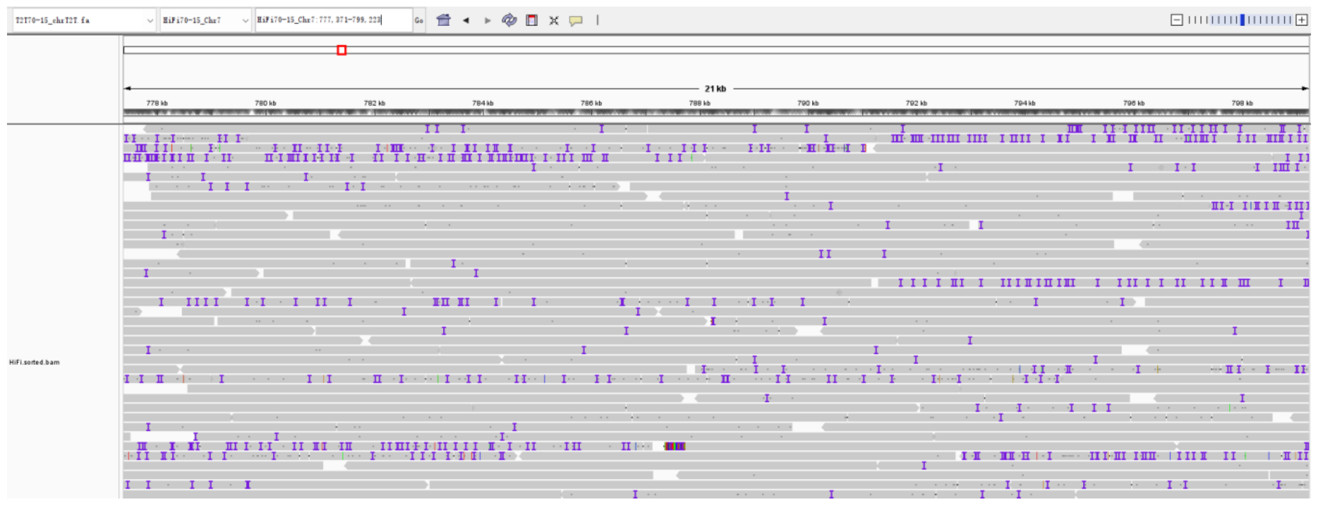

TRANS 10: HiFi70-15_Chr7:785,278-807,144

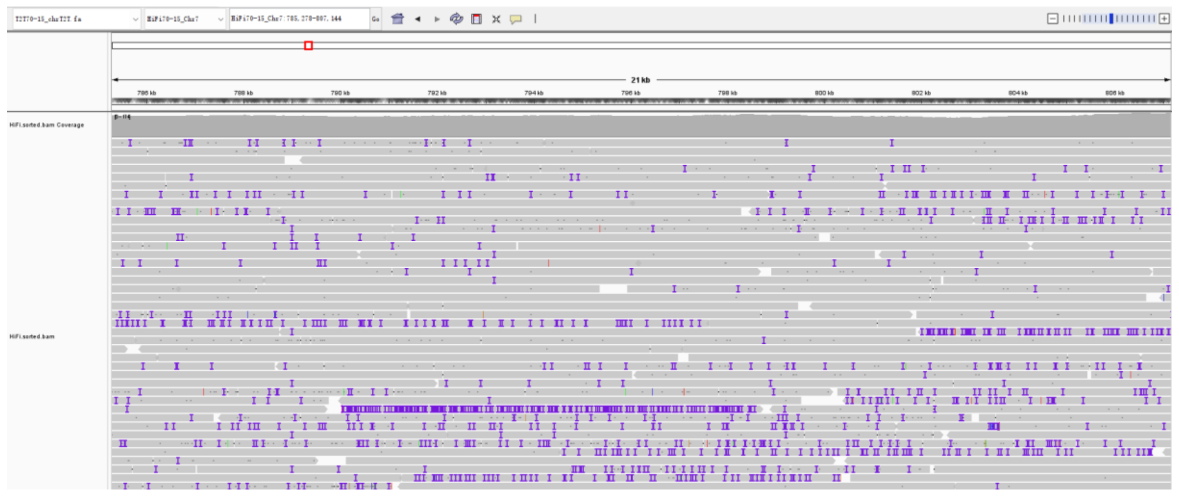

Fig. S3 Comparative analysis between the T2T 70-15 and MG8 genome assemblies identified the potential translocation.

The ten potential translocation regions were identified through alignment of the T2T 70-15 genome with the MG8 reference genome using SyRi. The validation of these structural variations, along with their flanking 10-kb genomic regions, was carried out by visualizing the coverage depth of HiFi reads mapping.

Figure S4

INV 1: HiFi70-15_Chr1:1,085,278-1,108,458

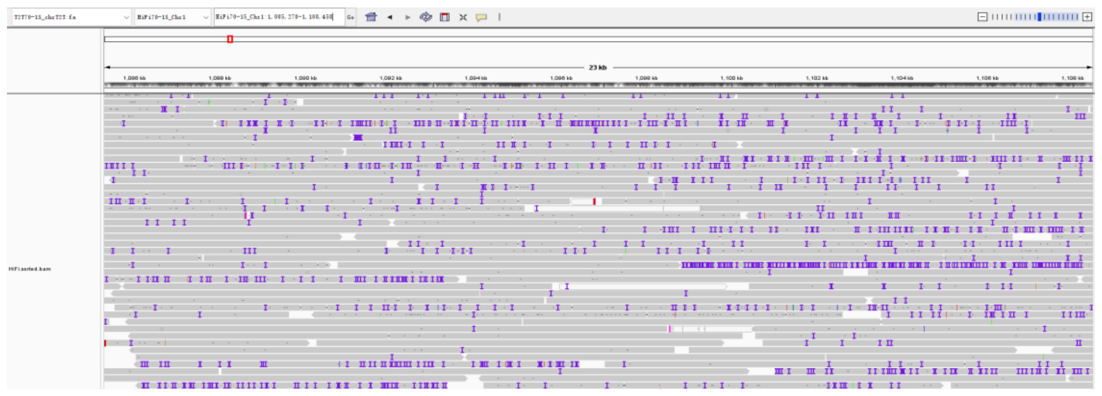

INV 2: HiFi70-15_Chr1:3,093,796-3,115,507

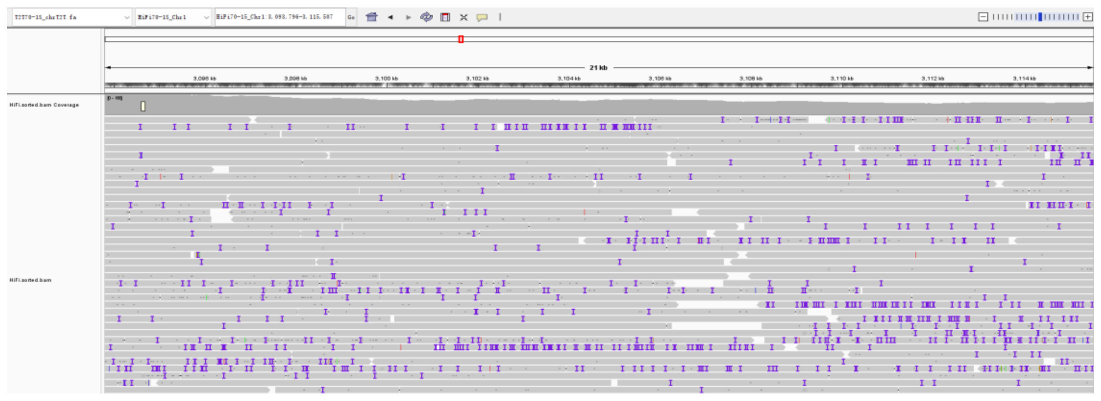

INV 3: HiFi70-15_Chr1:4,262,155-4,284,003

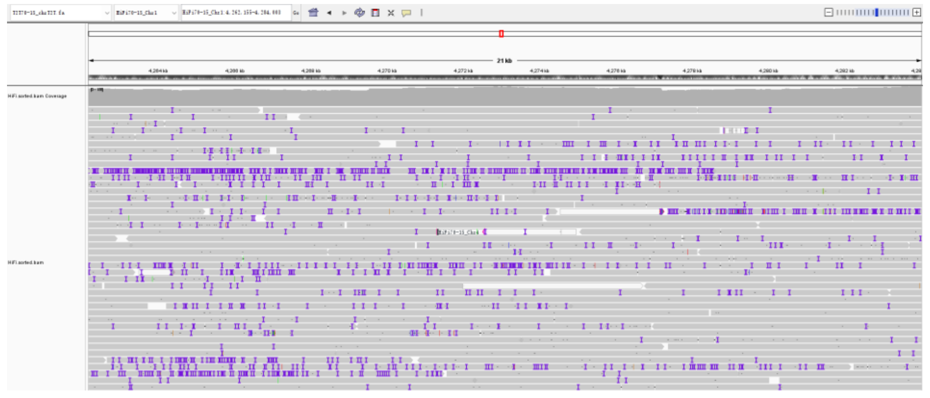

INV 4: HiFi70-15_Chr2:136,054-156,447

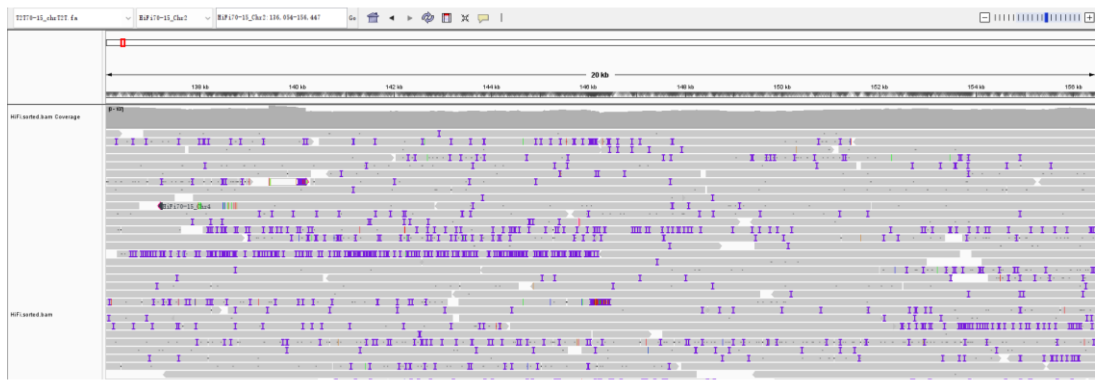

INV 5: HiFi70-15_Chr2:8,098,811-8,120,536

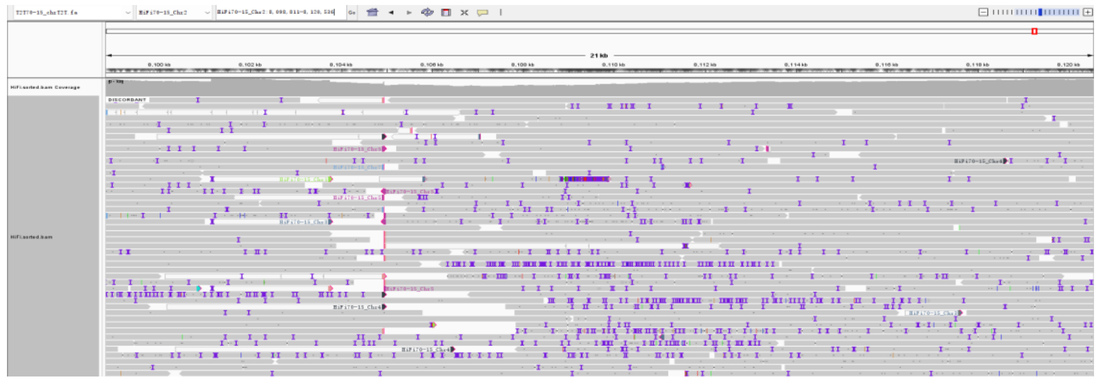

INV 6: HiFi70-15_Chr3:264,100-284,922

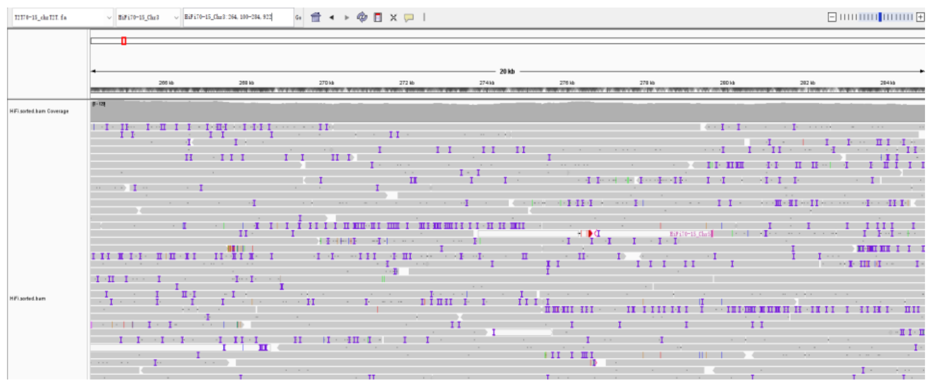

INV 7: HiFi70-15_Chr3:6,784,852-6,805,362

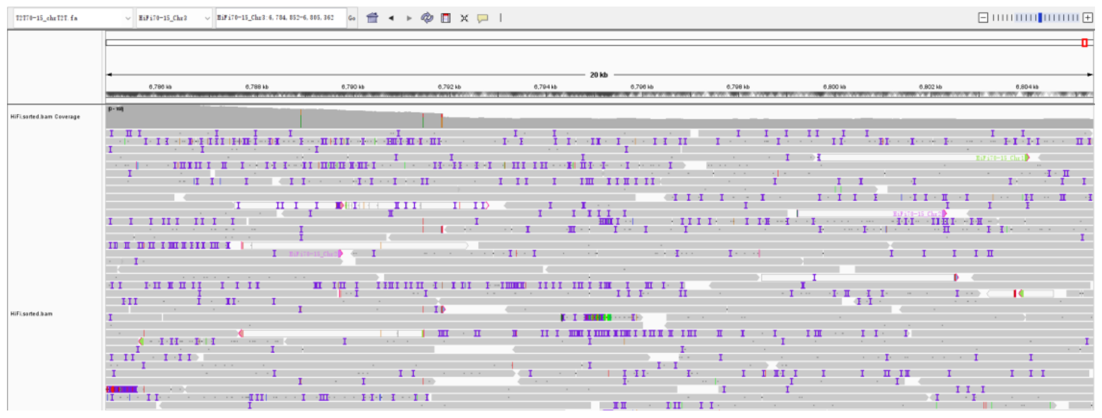

INV 8: HiFi70-15_Chr4:511,380-532,717

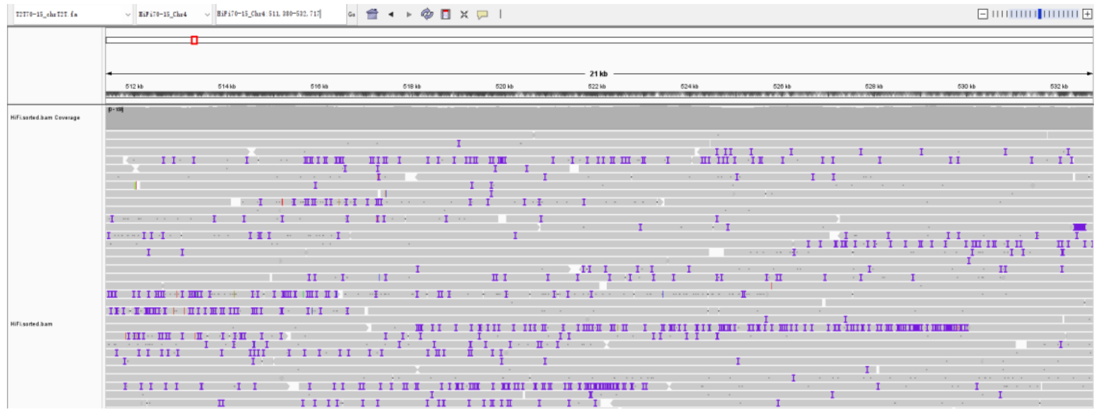

INV 9: HiFi70-15_Chr6:745,358-765,610

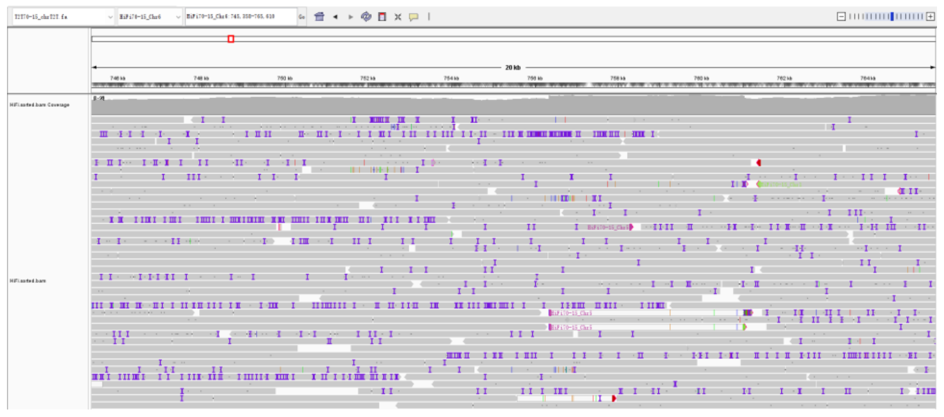

INV 10: HiFi70-15_Chr6:1,537,214-1,641,595

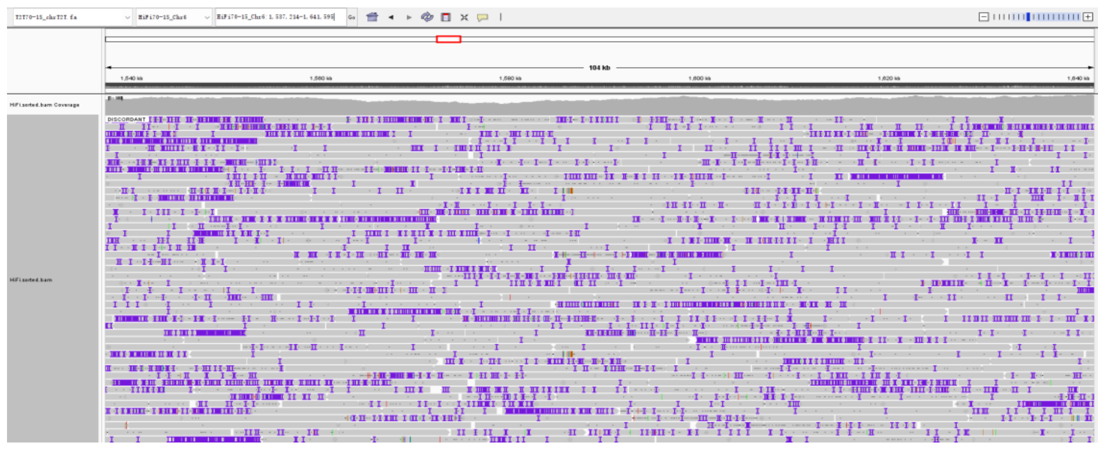

INV 11: HiFi70-15_Chr6:3,615,657-3,639,425

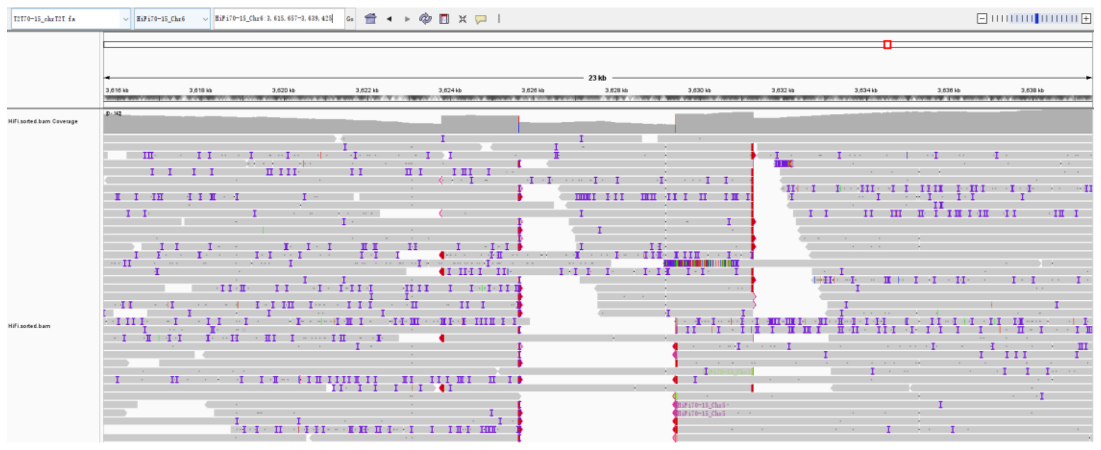

INV 12: HiFi70-15_Chr7:3,437,450-3,486,734

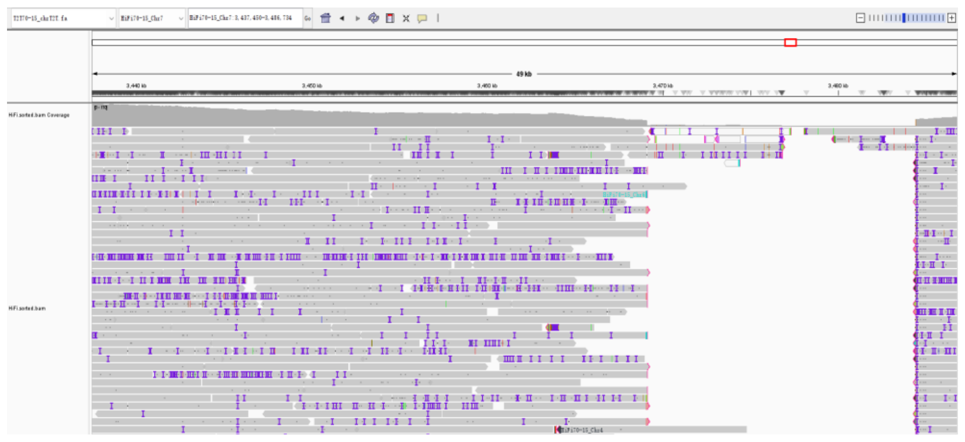

Fig. S4 Comparative analysis between the T2T 70-15 and MG8 genome assemblies identified the potential inversion.

The twelve potential inversion regions were identified through alignment of the T2T 70-15 genome with the MG8 reference genome using SyRi. The validation of these structural variations, along with their flanking 10-kb genomic regions, was carried out by visualizing the coverage depth of HiFi reads mapping. INV 11 and INV12 were located around the original gap region. Although these gaps could be resolved through gap filling using both next-generation and third-generation sequencing data, this process might introduce potential errors into the assembled genome.

Figure S5

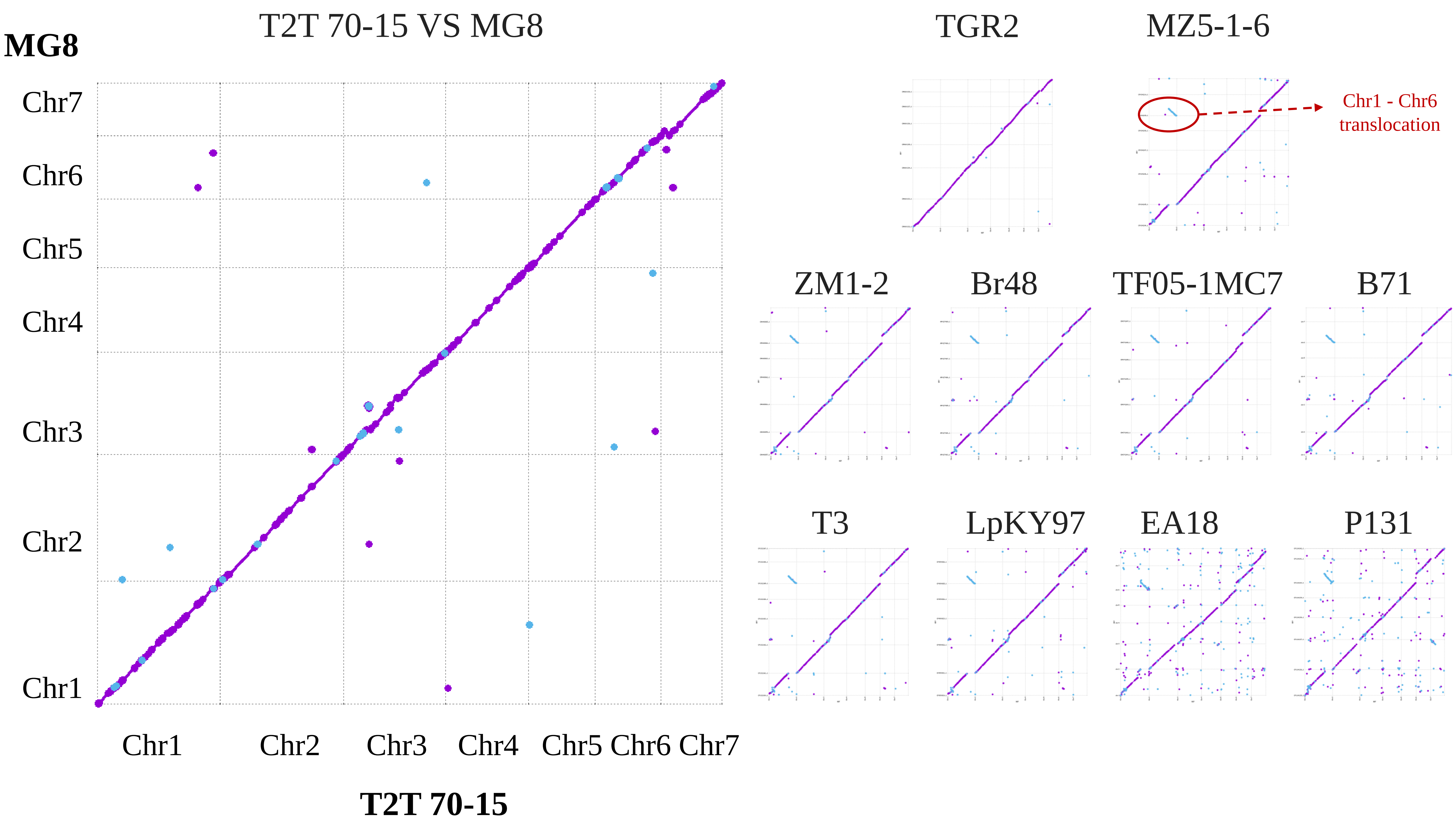

Fig. S5 Genomic-level collinearity analysis of T2T 70-15 with the *M. oryzae* species complex.

Ten public long-read sequencing genomes were collected to align with T2T 70-15. T2T 70-15 showed excellent collinearity with the MG8 version genome and almost no structural variation was observed (left). The previously reported large chromosomal translocation within the *M. oryzae* species at the boundary between chromosome 1 and chromosome 6 can also be detected (right).
